# Protocol for culture, drug treatment, and dissociation of patient-derived 3D ovarian cancer microspheroids for flow cytometry

**DOI:** 10.1016/j.xpro.2026.104480

**Published:** 2026-03-31

**Authors:** Mariam Haffa, Karolina Juhani, Greta Gudoityte, Rita Hutyra-Gram Ötvös, Emelie Wallin, Olli-Pekka Kallioniemi, Josefin Fernebro, Ulrika Joneborg, Brinton Seashore-Ludlow

**Affiliations:** 1 Science for Life Laboratory, Department of Oncology-Pathology, Karolinska Institutet, 17177 Solna, Sweden; 2 Department of Oncology-Pathology, Karolinska Institutet, 17177 Solna, Sweden; 3 Department of Pelvic Cancer, Theme Cancer, Karolinska University Hospital, 17176 Stockholm, Sweden; 4 Institute for Molecular Medicine Finland, University of Helsinki, 00290 Helsinki, Finland; 5 Department of Women’s and Children’s Health, Division of Obstetrics and Gynecology, Karolinska Institutet, 17177 Solna, Sweden

**Keywords:** Cancer, Cell Biology, Cell culture, Cell isolation, Cell-based Assays, Flow Cytometry, Single Cell

## Abstract

Single-cell technologies provide insights into tumor heterogeneity and treatment response. Here, we present a protocol to culture patient-derived ovarian tumor cells as 3D microspheroids. We describe steps for preparing multi-well dishes and isolating ovarian tumor cells for spheroid generation and culture. We then detail procedures for drug treatment, recovery, and dissociation of spheroids followed by single-cell analysis via flow cytometry. This scalable and cost-efficient protocol largely preserves the cellular composition of the original material.

## Highlights

• Protocol to culture patient-derived 3D tumor microspheroids for single-cell analysis
• Guidance on gentle recovery and dissociation of microspheroids into single cells
• Steps for drug response testing with single-cell level readout using flow cytometry

## Abstract (editor highlights)

Publisher’s note: Undertaking any experimental protocol requires adherence to local institutional guidelines for laboratory safety and ethics.

## Graphical abstract

**Figure:**
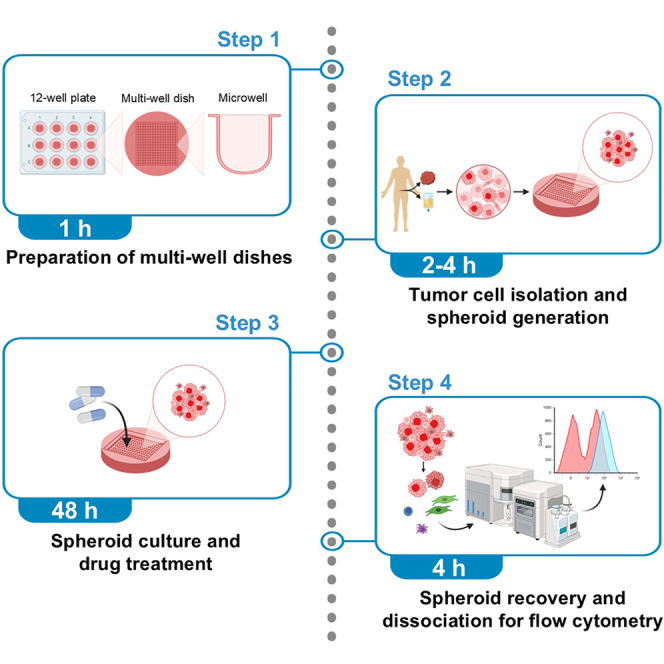

## Before you begin

### Rationale

Advances in single-cell technologies have revolutionized molecular and cell biology, provided exciting insights into tissue heterogeneity and cellular functions, and improved our understanding of disease progression and treatment responses, particularly in cancers.^1^ Single-cell studies have uncovered heterogenous cell populations in many solid tumors and hematologic malignancies and their role in treatment response.^2^^,^^3^ However, preserving the native microenvironment and cellular composition for *ex vivo* studies remains challenging. While traditional two-dimensional (2D) cell culture models are well-established and easily scalable, they fail to replicate the cellular complexity of *in vivo* tissues. In contrast, three-dimensional (3D) cell cultures mimic *in vivo* tissue architecture and the complex cell-cell and cell-matrix interactions more accurately,^4^ facilitating cellular organization, polarization, and differentiation, while maintaining the viability of different cell types, including rare cell populations.^5^ Moreover, the 3D structure of these cultures affects access to nutrients, oxygen, and drugs in a manner that closely resembles *in vivo* conditions.^6^^,^^7^^,^^8^^,^^9^ These physiologically relevant features are particularly important when studying cellular heterogeneity and function using single-cell methods. Single-cell experiments often require high cell number input, which can be sourced from 3D bulk cultures of patient-derived cells.^10^ However, these bulk cultures tend to form cell aggregates of varying size potentially affecting juxtacrine signaling, cell function, and responses to external stimuli such as drug treatment. In contrast to bulk cultures, microspheroids are small, uniform 3D cell models that offer improved control over aggregate size and high reproducibility. They are compatible with high-throughput screening, however, up-scaling for subsequent single-cell analysis is a challenge.

### Purpose of the protocol

In this protocol, we present a scalable technique for culturing hundreds to thousands of 3D microspheroids from patient-derived cells, including their recovery and dissociation into single-cell suspensions. Our protocol offers a flexible, cost- and time-efficient system utilizing recyclable molds and common reagents that has recently been presented for the characterization of 3D tumor models via confocal imaging.^11^^,^^12^ This method can be easily integrated into existing patient sample processing workflows. Using patient-derived ovarian tumor cells, we demonstrate that this technique produces microspheroids that reveal patient-specific morphology patterns. The dissociation protocol preserves the integrity of single cells regarding viability and surface marker expression. We show that the microspheroids largely retain the cellular composition of the original patient material, including rare cell populations. Additionally, we demonstrate that the protocol is suitable for investigating the mechanisms of action of compounds on a single-cell level. This protocol describes specific steps optimized for patient-derived ovarian tumor cells but can be adopted for cells from other tumor entities or other tissues.

### Innovation

This protocol introduces a scalable workflow for the simultaneous generation, culture, drug treatment and dissociation of hundreds to thousands of patient-derived 3D microspheroids. The protocol is optimized for downstream single-cell analyses, such as flow cytometry. Unlike conventional 3D bulk culture methods that produce heterogenous aggregates of variable size, microspheroids ensure improved control over size and uniformity, as well as consistent drug exposure across samples. Key advantages of the protocol are the flexible integration into existing patient sample workflows and the cost-efficient use of recyclable molds and commonly available reagents. It bridges the gap between compound testing in 3D *ex vivo* ovarian cancer models and single-cell readouts by maintaining the patient-specific cellular heterogeneity. The presented method can be flexibly adapted to other tissue types and single-cell readouts and thereby expands the repertoire of methods for single-cell analyses in translational cancer research and personalized drug testing.

### Institutional permissions

Patient samples were obtained from Karolinska University Hospital in Solna, Sweden. The study was approved by the Swedish Ethical Review Authority (2020-05830) and all patients provided written informed consent. An ethical permission from the relevant institution is required for any work with primary human samples described in this protocol.

### Preparation for multi-well dish production

**Timing: 3 h**

Different casting mold formats are available for the preparation of multi-well dishes. Here we describe the usage of the 12–256 casting mold (16 × 16 array).

1. Prepare a sterile 0.9 % NaCl solution.
  a. Weigh 9 g of NaCl powder in a dry autoclave-safe glass bottle.
  b. Add 1 L of sterile water to the NaCl powder.
  c. Autoclave the NaCl solution.
  d. Store at 20°C.
2. Prepare sterile agarose powder.
  a. Weigh 0.5 g agarose powder in a dry autoclave-safe glass bottle.
  b. Screw on the lid loosely, but make sure no steam can enter the bottle.
  c. Autoclave the agarose powder on dry cycle.
  d. Store at 20°C.
3. Prepare sterile casting molds.
  a. Put the casting molds into an autoclave-safe container, e.g., autoclaving pouches.
  b. Autoclave the casting molds on dry cycle.
4. Prepare Culture Medium according to the “materials and equipment” section in a sterile environment and prewarm at 37°C. After usage, store Culture Medium at 4°C for up to 2 weeks.
5. Prepare a 12-well culture plate with sterile tweezers or spatula.

### Preparation for tissue sample processing

**Timing: 30 min**

6. Prepare the dissociation enzymes according to the manufacturer’s protocol: https://static.miltenyibiotec.com/asset/150655405641/document_qlvs47ia0p6lv0mm12nmpm0i46?content-disposition=inline. Store the enzymes in ready-to-use aliquots at −20°C for up to 6 months.
7. Prepare Stop Medium according to the “materials and equipment” section in a sterile environment and prewarm to 37°C. After usage, store Stop Medium at 4°C for up to 1 month.
8. Prewarm basal DMEM medium (without any additives) to 37°C.
9. Prewarm Culture Medium to 37°C.
10. If cells are intended to be viably frozen: Prepare Freezing Medium according to the “materials and equipment” section in a sterile environment and prewarm an aliquot to 37°C. Store unused Freezing Medium in aliquots at −20°C for up to 1 month.
11. Prepare the following equipment:
  a. Pre-chilled Phosphate Buffer Saline (PBS).
  b. Petri dish.
  c. Sterile scalpel and tweezer.
  d. gentleMACS C Tubes.
  e. gentleMACS Dissociator.
  f. Rotator.
  g. 70 μm strainer.
  h. 50 mL tubes.
  i. ACK Lysis buffer 1X.
  j. Trypan blue solution.
  k. Cell counting equipment, such as automated cell counter.
  l. If cells are to be viably frozen: Labelled cryotubes.
  m. If cells are to be viably frozen: Cell freezing container.

### Preparation for ascites sample processing

**Timing: 20 min**

12. Prepare Stop Medium according to the “materials and equipment” section in a sterile environment and prewarm to 37°C. After usage, store Stop Medium at 4°C for up to 1 month.
13. Prewarm Culture Medium to 37°C.
14. If cells are intended to be viably frozen: Prepare Freezing Medium according to the “materials and equipment” section in a sterile environment and prewarm an aliquot to 37°C. Store unused medium in aliquots at −20°C for up to 1 month.
15. Prepare the following equipment:
  a. 50 mL tubes or larger centrifugation bottles (e.g., Thermo Scientific™ Polypropylene Centrifuge Bottles Catalog No. 75-006-443), including corresponding centrifugation inserts.
  b. 70 μm strainer.
  c. ACK Lysis buffer 1X.
  d. Trypan blue solution.
  e. Cell counting equipment, such as automated cell counter.
  f. If cells are to be viably frozen: Labelled cryotubes.
  g. If cells are to be viably frozen: Cell freezing container.

### Preparation for thawing viably frozen tumor cells

**Timing: 10 min**

16. Prewarm Culture Medium to 37°C.
17. Turn on a heat block with a cryotube adapter to 37°C.
18. Prepare dry ice if frozen cells need to be transported.
19. Prepare the following equipment:
  a. 50 mL tubes.
  b. Trypan blue solution.
  c. Cell counting equipment, such as automated cell counter.

### Preparation for recovery and dissociation of microspheroids

**Timing: 20 min**

20. Prepare Recovery Medium according to the “materials and equipment” section in a sterile environment and prewarm to 37°C. After usage, store Recovery Medium at 4°C for up to 1 month.
21. Prewarm Culture Medium to 37°C.
22. Prewarm an aliquot of required volume of TrypLE™ Express Enzyme 1X solution to 37 °C.
23. Turn on a heat block to 37°C.
24. Prepare the following equipment:
  a. Sterile tweezers or spatula.
  b. 2 mL dolphin tubes.
  c. 96-well flat-bottom plate (e.g., flat-bottom cell culture plate with standard surface, Sarstedt, Catalog No. 83.3924.005).
  d. 20 μm cell strainer.
  e. 50 mL tubes.
  f. Trypan blue solution.
  g. Cell counting equipment, such as automated cell counter.

### Preparation for enrichment of viable cells

**Timing: 5 min**

25. Prewarm Recovery Medium to 37°C.
26. Prepare the following equipment:
  a. Dead Cell Removal Kit.
  b. LS columns.
  c. QuadroMACS™ Separator.
  d. Sterile, double-distilled water.
  e. 15 mL tubes.
  f. 2 mL dolphin tubes.
  g. Optional: 20 μm mini strainer.

### Preparation for flow cytometry

**Timing: 30 min**

27. Prepare Flow Buffer according to the “materials and equipment” section in a sterile environment and keep at 4°C. After usage, store Flow Buffer at 4°C for up to 1 month.
28. Turn on a heat block to 67°C.
29. Prepare the following equipment:
  a. TruStain FcX.
  b. Markers needed (e.g., antibodies, caspase-3/7 marker and 7-AAD).
  c. 96-well V-shape microplate when using Attune N×T Flow Cytometer for data acquisition.
  d. Reaction tube 1.5 mL for handling the dead cell control cells.
  e. Start the flow cytometry instrument.

## Key resources table

**Table:** 

REAGENT or RESOURCE	SOURCE	IDENTIFIER
**Antibodies**		

Human TruStain FcX™ (FC Receptor Blocking Solution)	BioLegend	Cat#422302; RRID: AB_2818986
anti-human CD326-APC (EpCAM), clone: 9C4 (1:50 dilution)	BD Bioscience	CAT#566842; RRID: AB_2869899
anti-human CD45-PE-Cy7, clone: HI30 (1:50 dilution)	BD Bioscience	CAT#560915; RRID: AB_396854
anti-human CD90-PE, clone: 5E10 (1:50 dilution)	BD Bioscience	CAT#555596; RRID: AB_395970
anti-human PDPN-BV605, clone: LpMab-17 (1:50 dilution)	BD Bioscience	CAT#747634; RRID: AB_2744199
anti-human CD31-BV421, clone: MBC 78.2 (1:50 dilution)	BD Bioscience	CAT#744074; RRID: AB_2741976
anti-human CD45-BV421, clone: HI30 (1:50 dilution)	BD Bioscience	CAT#563879; RRID: AB_2744402

**Biological samples**		

Resected tumor samples from patients with high-grade serous ovarian cancer	Karolinska University Hospital in Solna, Sweden	N/A
Ascites fluid samples from patients with high-grade serous ovarian cancer	Karolinska University Hospital in Solna, Sweden	N/A

**Chemicals, peptides, and recombinant proteins**		

Agarose, low gelling temperature	Sigma-Aldrich	Cat#A9414
DMEM, no glucose, no L-glutamine, no phenol red	Thermo Fisher Scientific	Cat#A1443001
Ham’s Nutrient Mixture F-12	Thermo Fisher Scientific	Cat#11968075
B-27 serum-free supplement 50X	Thermo Fisher Scientific	Cat#17504001
L-Glutamine 200 mM Cytiva	Thermo Fisher Scientific	Cat#SH30034.01
HEPES 1 M	Thermo Fisher Scientific	Cat#15630056
HyClone™ Pencillin-Streptomycin 100X Solution	Thermo Fisher Scientific	Cat#SV30010
Recombinant human EGF	Peprotech	Cat#AF-100-15
Recombinant human FGF-basic	Peprotech	Cat#AF-100-18B
ACK Lysis buffer 1X	Thermo Fisher Scientific	Cat#A1049201
Trypan blue solution	Thermo Fisher Scientific	Cat#25-900-CI
Cisplatin	Sigma-Aldrich	Cat#232120-50MG
7-Amino-Actinomycin (7-AAD)	BD Bioscience	Cat#559925
CellEvent™ Caspase-3/7 Green Detection Reagent	Thermo Fisher Scientific	Cat#C10423

**Critical commercial assays**		

MicroTissues® 3D Petri Dish® micro-mold spheroids 16 × 16 array (12-256 cast mold)	Sigma Aldrich	Cat#Z764000
12-well cell culture plate, flat-bottom, surface: standard	Sarstedt	Cat#83.3921
Tissue culture dish, diameter: 10 cm, surface: standard	Sarstedt	Cat#83.3902
Tumor Dissociation Kit, human	Miltenyi	Cat#130-095-929; RRID:SCR_020276
gentleMACS™ C Tubes	Miltenyi	Cat#130-093-237; RRID:SCR_020270
gentleMACS™ Dissociator	Miltenyi	Cat#130-093-235; RRID:SCR_020267
Cell strainer pluriStrainer 70 μm pore size	pluriSelect	Cat#43-50070-51
Cell strainer pluriStrainer 20 μm pore size	pluriSelect	Cat#43-50020-03
Mini cell strainer pluriStrainer 20 μm mesh size	pluriSelect	Cat#43-10020-50
Mini cell strainer pluriStrainer 40 μm mesh size	pluriSelect	Cat#43-10040-50
Cryotubes 2 mL, QuickSeal screw cap	Sarstedt	Cat#72.380.992
CoolCellTM LX Freezing Container	Merck	Cat#BCS-405G
2 mL dolphin tubes	Merck	Cat#CLS3213
TrypLE™ Express Enzyme 1X	Thermo Fisher Scientific	Cat#11538856
Dead Cell Removal Kit	Miltenyi	Cat#130-090-101
LS columns	Miltenyi	Cat#130-042-401
QuadroMACS™ Separator	Miltenyi	Cat#130-090-976
96-well V-shape microplate	Greiner	Cat#651201
96-well cell culture plate, flat-bottom, surface: standard	Sarstedt	Cat#83.3924.005
Reaction tube 1.5 mL	Sarstedt	Cat#72.706.200

**Software and algorithms**		

FlowJo®	BD Life Sciences	RRID:SCR_008520https://www.flowjo.com/flowjo/download

**Other**		

Attune NxT Flow Cytometer	Thermo Fisher	RRID:SCR_019590

## Materials and equipment

### Recipes for culture media, buffers, and solutions

**Table:** Culture Medium

Reagent	Final concentration	Amount
DMEM, no glucose, no L-glutamine	N/A	236.25 mL
Nutrient Mixture F-12, with 1 mM L-glutamine	N/A	236.25 mL
B27 supplement 50X	1X	10 mL
L-Glutamine 200 mM	2.5 mM∗	5 mL
HEPES 1 M	15 mM	7.5 mL
Penicillin-Streptomycin 100X	1X	5 mL
EGF 1 mg/mL	20 ng/mL	10 μL
Basic FGF 0.5 mg/mL	10 ng/mL	10 μL
**Total**	**N/A**	**500 mL**

Reconstitute EGF lyophilizate with 10 mM acetic acid in sterile water to get a solution of 1 mg/mL. Reconstitute basic FGF lyophilizate with 5 % trehalose in sterile water to get a solution of 0.5 mg/mL. Store EGF and FGF aliquots at −20°C for up to 12 months. Mix all components of the Culture Medium under sterile conditions, store at 4°C for up to 2 weeks.

∗Total L-Glutamine concentration.

***Note:*** The Culture Medium described here is optimized for patient-derived ovarian tumor spheroid cultures.^13^ Alternative medium formulations optimized for the cells under study may be used.

**Table:** Stop Medium

Reagent	Final concentration	Amount
DMEM medium	N/A	500 mL
FBS	N/A	50 mL
Pencillin-Streptomycin 100X	1X	5 mL
**Total**	**N/A**	**550 mL**

Mix all components under sterile conditions, store at 4°C for up to 1 month.

#### Freezing medium 1X

• Weigh 149 g of methyl cellulose in a dry autoclave-safe glass bottle.
• Autoclave methyl cellulose on dry cycle.
• Add 40 mL FBS.
• Stir for 1 h.
• Add 22.2 mL FBS.
• Add 11.85 mL DMSO.
• Mix Freezing Medium with Culture Medium 1:1 (v/v).
• Store in aliquots at −20°C for up to 1 month.

#### Recovery medium

• Weigh 2 g of BSA in a dry glass bottle.
• Add 100 mL Culture Medium.
• Keep at 4°C for 12 – 24 h to dissolve BSA.
• Filter through a bottle-top sterile filter unit of 0.2 μm pore size.
• Store at 4°C for up to 1 month.

#### Flow buffer

• Add 400 μL of 0.5 M EDTA to 100 mL PBS to get a 2 mM solution.
• Weigh 0.5 g of BSA in a dry glass bottle.
• Add 2 mM EDTA solution to BSA.
• Keep at 4°C for 12 – 24 h to dissolve BSA.
• Filter through a bottle-top sterile filter unit of 0.2 μm pore size.
• Store at 4°C for up to 1 month.

## Step-by-step method details

### Preparation of multi-well dishes

**Timing: 1 h for six casting molds**

The first section of the protocol describes the preparation of the multi-well dishes according to the manufacturer’s protocol: https://www.microtissues.com/protocols.

***Note:*** The agarose multi-well dishes can be prepared one day in advance and stored in Culture Medium in a humidified incubator.

1. Prepare a 2 % (w/v) agarose solution by adding 25 mL sterile NaCl 0.9 % solution to 0.5 g autoclaved agarose powder.
2. Gently swing the bottle to premix the powder.
3. Place the bottle in a microwave with the lid slightly open and heat the solution until the agarose powder has completely dissolved.
  a. Check every 10 to 15 seconds to avoid overboiling.
  b. Depending on the microwave, dissolving the agarose powder can take around 1 min.
  c. No translucent, undissolved agarose particles should be visible.
    ***Note:*** The agarose solution and glass bottle can get very hot. Touch the bottle only with heat-resistant gloves.
4. Let the agarose solution cool down to around 60 – 70°C. This takes around 5–10 min.
5. Put the casting molds on the bench with the indentation facing upwards (Figure 1A).

**Figure 1:**
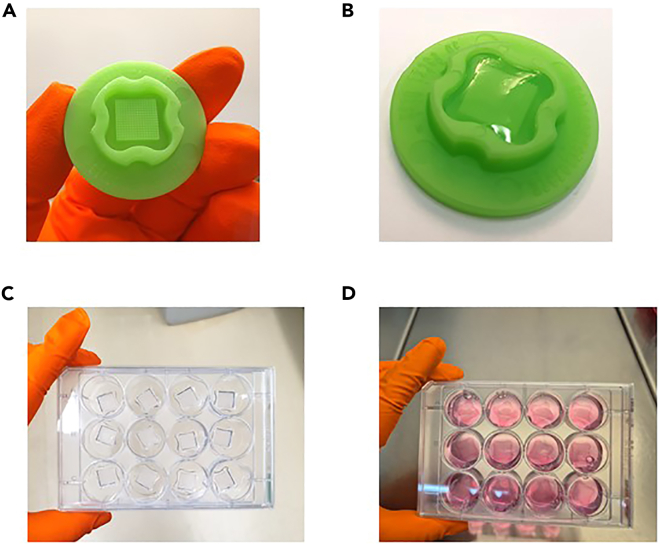
Preparation of multi-well dishes (A) Empty casting mold. (B) Casting mold filled with gelled agarose. (C) Multi-well dish released into a 12-well plate. (D) Multi-well dishes in medium.
6. Slowly pipette 500 μL of molten agarose into each casting mold.
  a. Avoid creating bubbles.
  b. Remove any bubbles that are trapped in the protrusions of the mold by pipetting or gentle tapping.
    ***Note:*** It is difficult to pipette agarose that has cooled down too much. The cool agarose solution can be re-heated in this case.
7. Keep the agarose-filled molds at 20°C for 20 – 30 min until the agarose has gelled (Figure 1B).
8. Release the agarose dishes into the wells of a 12-well plate by inverting and carefully flexing the mold. Use a sterile tweezer or spatula for assistance. Each well of the 12-well plate should contain one agarose dish (Figure 1C). Problem 1.
  **CRITICAL:** The agarose multi-well dishes can easily break during this step. Some practice might be necessary to release the dishes. Extra agarose multi-well dishes can be prepared to make sure enough undamaged dishes are available for the experiment.
  ***Note:*** An agarose solution of 25 mL is sufficient to prepare around 40 dishes. The agarose solution can be re-heated several times to prepare additional sets of multi-well dishes.
9. Add 2–2.5 mL of Culture Medium to each well of the 12-well plate that contains a multi-well dish.
10. Incubate for at least 15 min.
11. Remove Culture Medium surrounding the outside of the multi-well dishes.
12. Add 2–2.5 mL of Culture Medium to each well.
13. Incubate for at least 15 min.
14. Remove Culture Medium surrounding the outside of the multi-well dishes.
15. Add 2–2.5 mL of Culture Medium to each well (Figure 1D).
16. Store in a humidified incubator at 37°C until usage.

### Isolation of tumor cells from tissue samples

**Timing: 2–3 h depending on sample size**

This section describes the isolation of tumor cells from ovarian tumor tissue. Tumor tissue samples are dissociated using the Tumor Dissociation Kit, human according to the manufacturer’s protocol: https://static.miltenyibiotec.com/asset/150655405641/document_qlvs47ia0p6lv0mm12nmpm0i46?content-disposition=inline. The protocol is optimized for tumor cell isolation from ovarian cancer samples^14^ and can be adopted for isolating cells from other tissues or tumor entities.

**CRITICAL:** Start this procedure immediately after surgical resection or receipt of the tissue sample to ensure optimal sample viability.

17. Transfer the tissue to a petri dish.
18. Wash the tissue with pre-chilled PBS.
19. Remove non-malignant tissue, such as adipose and connective tissue, as much as possible.
20. Mince the tissue into 1 – 5 mm pieces with a disposable sterile scalpel.
21. Transfer the minced pieces to a gentleMACS C Tube and add 4.7 mL basal DMEM medium (without any additives), 200 μL of Enzyme H, 100 μL of Enzyme R, and 25 μL of Enzyme A.

***Note:*** Divide large samples into several dissociation tubes. Each tube should contain a maximum of 1 g minced tumor tissue.

22. Attach the C Tube upside down into the sleeve of a gentleMACS Dissociator and run program h_tumor_01 and h_tumor_02.
23. Put the C Tube into a rotator at 37°C at 300 rpm for 1 h.
24. Attach the C Tube upside down into the sleeve of a gentleMACS Dissociator and run program h_tumor_03.
25. Filter the sample through a 70 μm strainer and collect flow-through in a 50 mL tube.
26. Wash the strainer with 45 mL Stop Medium.
27. Centrifuge at 500 × *g* for 5 min at 20°C.
28. Discard the supernatant.

***Optional:*** If the cell pellet looks red, remove red blood cells as described in the section red blood cell removal (optional).

29. Resuspend the cell pellet in 10 mL Culture Medium.
30. Count viable cells with trypan blue staining.

***Optional:*** If freshly isolated tumor cells cannot be directly used for microspheroid culture, cells can be viably frozen as described in the section viable cryopreservation of tumor cells (optional) and used at a later timepoint.

### Red blood cell removal (optional)

**Timing: 15–30 min**

This procedure removes red blood cells from the isolated tumor cell suspension. It can be applied when the cell pellet in step 28 (tissue sample) or step 44 (ascites sample) looks red.

31. Resuspend the cell pellet with 1 – 5 mL (depending on the pellet size) ACK Lysis buffer 1X.
32. Incubate for 5 min at 20°C.
33. Add 10 mL Stop Medium.
34. Centrifuge at 500 × *g* for 5 min.
35. Discard the supernatant.
36. Repeat steps 31 – 35, if the cell pellet still looks red.

### Viable cryopreservation of tumor cells (optional)

**Timing: 5 min**

This section describes the procedure for viable cryopreservation of tumor cells when freshly isolated cells cannot be used immediately for microspheroid culture.

37. Resuspend cells in 1X Freezing Medium by adding 800 – 1000 μL Freezing Medium to 3 – 6 million cells.
38. Transfer the cell suspension to a labelled cryotube.
39. Freeze cells at – 80°C using a cell freezing container.

### Isolation of tumor cells from ascites samples

**Timing: 1–2 h depending on sample size**

This section describes the isolation of tumor cells from ascites fluid collected from ovarian cancer patients.^14^ This part of the protocol can be adopted for isolating cells from other fluids or tumor entities.

**CRITICAL:** Start this procedure immediately after collection or receipt of the ascites sample.

40. Cool down the centrifuge to 4°C.
41. Transfer ascites to 50 mL tubes.
42. Centrifuge at 800 × *g* for 10 min at 4°C.

***Optional:*** If the ascites volume is very large, ascites can be transferred to larger centrifuge bottles. For example, Thermo Scientific™ Polypropylene Centrifuge Bottles (Catalog No. 75-006-443) can be used. Make sure to use the right centrifuge inserts for the centrifugation bottles.

43. Remove the supernatant.

***Optional:*** The supernatant contains acellular components, such as metabolites, soluble proteins and exosomes and can be stored at – 80°C for later use.

44. Pool all cell pellets.

***Optional:*** If the cell pellet looks red, remove red blood cells as described in the section red blood cell removal (optional).

45. Resuspend the cell pellet in 10 mL Stop Medium.
46. Filter the sample through a 70 μm strainer and collect the flow-through in a 50 mL tube.
47. Wash the strainer with up to 40 mL Stop Medium.
48. Centrifuge at 500 × *g* for 5 min at 20°C and discard the supernatant.
49. Resuspend the cell pellet in 10 mL Culture Medium.
50. Count viable cells with trypan blue staining.

***Optional:*** If freshly isolated tumor cells cannot be directly used for microspheroid culture, cells can be viably frozen as described in the section viable cryopreservation of tumor cells (optional) and used at a later timepoint.

### Thawing viably frozen patient-derived tumor cells

**Timing: 30 min**

As an alternative to using freshly isolated patient-derived tumor cells for microspheroid culture, viably frozen patient-derived tumor cells can be used. Note that the fraction of dead cells can be higher for frozen cells compared to fresh cells.

51. Prewarm 20 mL of Culture Medium in a 50 mL tube at 37°C.
52. Get viably frozen cells from the freezer or liquid nitrogen. Make sure cells are transported on dry ice.
53. Quickly thaw cells using the heat block.
54. Immediately transfer the cells to the Culture Medium prepared in step 51.

**CRITICAL:** Steps 53 and 54 should be performed very quickly, as cells are very sensitive to DMSO.

55. Centrifuge at 200 × *g* for 5 min at 20°C.
56. Remove the supernatant.
57. Resuspend cells in 10 mL Culture Medium.
58. Count viable cells with trypan blue staining.

### Seeding tumor cells to generate microspheroids

**Timing: 1 h for one sample and 20 multi-well dishes**

This section describes the generation of microspheroids in the agarose multi-well dishes. For this, either cells that are freshly isolated from tumor tissue or ascites fluid, or cells that have been viably frozen can be used. Note that the fraction of dead cells can be higher for frozen cells compared to fresh cells.

59. Adjust the concentration of cells according to Table 1.

**Table 1:** Cell concentrations for generating different sized microspheroids

Cells per microspheroid	Number of cells per microwell dish (for 256 spheroids)	Cell concentration
100	25 600 in 100 μL	2.56 × 10ˆ5/mL
200	51 200 in 100 μL	5.12 × 10ˆ5/mL
400	102 400 in 100 μL	1.02 × 10ˆ6/mL
600	153 600 in 100 μL	1.54 × 10ˆ6/mL
800	204 800 in 100 μL	2.05 × 10ˆ6/mL
1 000	256 000 in 100 μL	2.56 × 10ˆ6/mL
60. Remove Culture Medium surrounding the outside of the multi-well dishes (Figure 2A).

**Figure 2:**
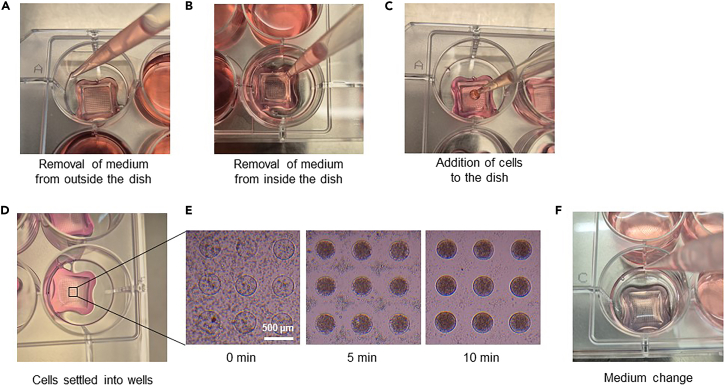
Seeding of tumor cells (A) Medium removal from outside the multi-well dish. (B) Medium removal from inside the multi-well dish. (C) Addition of cells to the multi-well dish. (D) Cells settled into the wells of the dish. (E) Exemplary images of cells settled into wells. Scale bar = 500 μm. (F) Medium change.

**CRITICAL:** The following steps should be performed very carefully as multi-well dishes can easily break if moved roughly.

61. Carefully remove the Culture Medium from the cell seeding chamber of all multi-well dishes by tilting the culture plate (Figure 2B).
62. Carefully add 100 μL of cell suspension dropwise into the cell seeding chamber of each multi-well dish (Figure 2C).
63. Let the cells settle into the multi-wells for 5 to 10 min (Figure 2D). Check the cells under the microscope (Figure 2E).
64. Add 2–2.5 mL Culture Medium to the outside of the multi-well dishes.
65. Incubate in a humidified incubator at 37°C.
66. Change medium every 3 to 4 days (Figure 2F).
  a. Remove Culture Medium surrounding the outside of the multi-well dishes.
  b. Add 2–2.5 mL of fresh Culture Medium to each well.
    ***Note:*** The morphology and size of microspheroids varies between patient samples and depends on initial seeding density. Problem 2.

### Cisplatin treatment

**Timing: 49 h (1 h for 5 multi-well dishes + 48 h for treatment)**

This section describes the drug treatment of microspheroids. Here, we use cisplatin at a concentration of 50 μM.

***Note:*** Depending on the purpose of the experiment, cells need to be pre-cultured before drug addition to allow for sufficient time to form microspheroid structures. The length of pre-culture depends on the cells and can vary between samples. For patient-derived ovarian cancer cells, we recommend 1 to 3 days of pre-culture.

**CRITICAL:** Make sure to follow the institutional safety measures when working with cytotoxic drugs.

**CRITICAL:** Cisplatin solutions are unstable and should always be freshly prepared. Do not use DMSO to prepare the solution. Here, we describe the usage of sterile distilled water to prepare the cisplatin stock solution.

67. Prepare the cisplatin stock solution of 1 mM (20X).
  a. Weigh 3.01 mg of cisplatin.
  b. Dilute in 10 mL of sterile distilled water.
68. Prepare the cisplatin working solution of 50 μM.
  a. Mix 500 μL of cisplatin stock solution (20X) with 9.5 mL of fresh Culture Medium to make 10 mL of working solution.
69. Remove Culture Medium surrounding the outside of the multi-well dishes.
70. Add 2 mL of cisplatin working solution to the corresponding multi-well dishes.
71. Incubate the microspheroids in a humidified incubator at 37°C for 48 h.

### Recovery and dissociation of microspheroids

**Timing: 2–2.5 h**

This section describes the recovery of microspheroids for dissociation into single-cell suspensions and the enzymatic dissociation of cultured microspheroids into single cells. Problem 3

**CRITICAL:** If the microspheroids have been treated with drugs and the dead cell fraction needs to be further analyzed (e.g., by cell death assay), keep the number of washing steps at minimum to avoid washing away dead cells.

**Figure 3:**
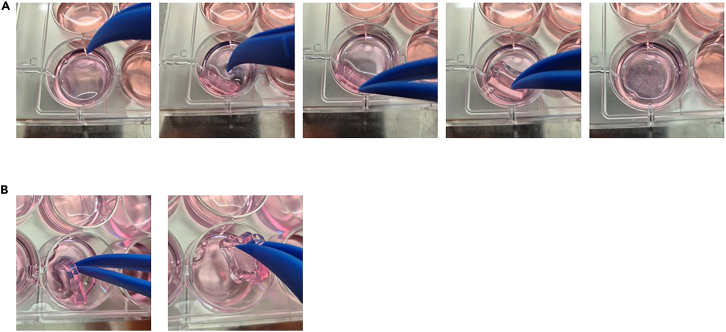
Release of microspheroids from multi-well dishes (A) Inverting a multi-well dish to release the microspheroids. (B) Removing a multi-well dish.

***Note:*** Agarose dishes can easily break during inverting. It is not critical if the dishes break into smaller pieces during this step, but the pieces should be large enough to be able to remove them.

72. Keep the multi-well dishes at 20°C for 10 min before continuing. Multi-well dishes are less fragile at 20°C.
73. Remove Culture Medium surrounding the outside of the multi-well dishes (Figure 2A).
74. Add 1 mL of Culture Medium to the outside of the multi-well dishes.
75. Invert the multi-well dishes within the same well by gently sliding a sterile tweezer or spatula underneath the dish, lifting it to an upright position and then carefully turning it over (Figure 3A). Problem 4.

76. Remove any bubbles that are trapped between the dish and the culture plate by pipetting or gentle tapping.
77. Centrifuge at 500 × *g* for 5 min.
78. Check if spheroids were released from the multi-well dishes under the microscope.
79. Remove the multi-well dishes by gently sliding a sterile tweezer or spatula underneath the dish, lifting it to an upright position and then carefully removing it from the well (Figure 3B).
80. Pool microspheroid suspensions of same condition.

***Optional:*** If the single-cell analysis method requires a high ratio of viable cells, dead cells can be removed from the microspheroid suspension by reverse filtering. Check the microspheroids under the microscope to determine if microspheroids are still intact and reverse filtering is possible. Do not reverse filter when the microspheroids are too small. Use a 20 μm strainer depending on the diameter of the microspheroid. Put the strainer onto a 50 mL tube and wet the strainer with 50 μL Culture Medium. Filter the spheroid suspension. Invert the strainer and put it onto a new 50 mL tube. Wash the strainer with around 5 mL Culture Medium and collect the spheroids in the flow-through.

81. Centrifuge at 500 × *g* for 5 min.
82. Remove supernatant and resuspend microspheroids in 1.5 mL Culture Medium.
83. Transfer the spheroid suspension to a 2 mL dolphin tube.
84. Centrifuge at 100 × *g* for 1 min.
85. Remove as much as possible of the supernatant.
86. Gently resuspend the spheroids in 1 mL of prewarmed TrypLE by pipetting.
87. Incubate the cells in the heat block at 37°C at 300 rpm for 15 to 30 min.
88. Check dissociation after 10 min and then after every 5 min by transferring 5 μL of cells to 96-well culture plate and inspecting under the microscope.

**CRITICAL:** Each patient sample forms distinct microspheroid structures that differ in tightness. Thus, dissociation time might highly vary between patient samples and tissue type. For ovarian cancer cells, we recommend 15 to 30 min. Longer incubation times might affect cell viability. Problem 5

89. Add 1 mL prewarmed Recovery Medium to the cell suspension to stop dissociation.
90. Gently mix the cells by pipetting.
91. Centrifuge at 300 × *g* for 5 min.
92. Remove the supernatant.
93. Gently resuspend the cell pellet in 200 μL Recovery Medium by pipetting.
94. Count viable cells and check for cell clumps. Problem 6.

***Optional:*** If the cell suspension contains large cell clumps, the suspension can be filtered through a 40 μm mini cell strainer. Put the strainer onto a 1 mL tube and wet the strainer with 50 μL Recovery Medium. Filter the cell suspension and collect the cells in the flow-through. Wash the strainer with around 200 μL Recovery Medium. Centrifuge the cells at 300 × *g* for 5 min and remove the supernatant. Resuspend the cells 1 mL Recovery Medium and transfer to 2 mL dolphin tube.

### Enrichment for viable cells (optional)

**Timing: 1–1.5 h**

If required, cell suspensions can be enriched for viable cells using the Dead Cell Removal Kit according to the manufacturer’s protocol: https://static.miltenyibiotec.com/asset/150655405641/document_9ndq9pouph0pvf6lmr5osgv41s?content-disposition=inline.

***Note:*** Skip this section, if characterization of dead cells after drug treatment is essential for downstream analysis.

95. Centrifuge cells at 300 × g for 5 min.
96. Remove the supernatant.
97. Gently resuspend the cells in 100 μL of Dead Cell Removal Microbeads by pipetting.
98. Incubate for 15 min at 20°C.
99. Prepare 1X Binding Buffer from 20X Binding Buffer Stock. Dilute with sterile, double-distilled water to prepare 15.5 mL 1X Binding Buffer per sample.

***Note:*** Depending on the column type, different volumes of Binding Buffer are needed. In this protocol, we use LS columns. Please refer to the manufacturer’s protocol for different column types: https://static.miltenyibiotec.com/asset/150655405641/document_9ndq9pouph0pvf6lmr5osgv41s?content-disposition=inline.

100. Place the column in the MACS Separator.
101. Rinse the column with 3 mL of 1X Binding Buffer.
102. Add 400 μL of 1X Binding Buffer to the cell suspension.
103. Transfer the cell suspension onto the column and collect the flow-through in a 15 mL tube (contains viable cells).
104. Wash the column four times with 3 mL of 1X Binding Buffer per washing step. Wait until the column reservoir is empty before starting the next washing step.
105. Collect the flow-through in the same 15 mL tube.
106. Centrifuge the flow-through at 300 × *g* for 5 min at 20°C.
107. Remove the supernatant.

***Optional:*** To increase efficiency of the enrichment, repeat steps 100 to 107 with a new column.

108. Resuspend the cells in 200 μL of Recovery Medium.
109. Transfer the cells to a 2 mL dolphin tube.
110. Count viable cells and check for cell clumps.

***Optional:*** To remove cell clumps, filter the cell suspension through a 20 μm mini strainer into a 2 mL dolphin tube.

### Analysis of cell composition via flow cytometry

**Timing: 2 h**

This section describes the analysis of single cells dissociated from microspheroids using flow cytometry. Here, we characterize the most abundant cell types present in ovarian tumor samples using a panel of specific antibodies as listed in Table 2. We use the Attune NxT Flow Cytometer for data acquisition. Adjust the protocol accordingly for other flow cytometer instruments.

111. Centrifuge cells at 300 × *g* for 5 min.
112. Remove supernatant.
113. Resuspend cells in 500 μL Flow Buffer.
114. Add 5 μL TruStain FcX and gently mix by pipetting.
115. Incubate for 10 min at 20°C.
116. Divide the sample into 50 μL aliquots and transfer one aliquot of each flow sample to a 96-well V-shaped microplate. For the antibody panel described here, prepare nine flow sample wells:
  a. One sample with all five antibodies and 7-AAD (7-AAD is added later).
  b. Six FMO controls: one control for each of the five antibodies and one control for 7-AAD.
  c. One dead cell control.
  d. One unstained control.
117. Prepare the 1:50 antibody dilutions with Flow Buffer according to Table 3.

**Table 3:** Antibody dilutions for flow cytometry

Flow sample	Volume per antibody	Volume flow buffer	Total volume
All five antibodies: CD326, CD45, CD90, PDPN, CD31	1.2 μL	54 μL	60 μL
FMO controls with four antibodies	1.2 μL	55,2 μL	60 μL
FMO control for 7-AAD with five antibodies	1.2 μL	54 μL	60 μL
118. Centrifuge the plate at 500 × *g* for 5 min.
119. Firmly flip the plate to remove the supernatant.
120. Add 50 μL of each antibody dilution to the cells and add Flow Buffer to the dead cell control and to the unstained control.
121. Gently mix by pipetting up and down.
122. Incubate for 20 min at 4°C in the dark.
123. Add 100 μL Flow Buffer to the cells.
124. Centrifuge the plate at 500 × *g* for 5 min.
125. Firmly flip the plate to remove the supernatant.
126. Resuspend the cells in 100 μL Flow Buffer.
127. Centrifuge the plate at 500 × *g* for 5 min.
128. Firmly flip the plate to remove the supernatant.
129. Resuspend the cells in 100 μL Flow Buffer.
130. Centrifuge the plate at 500 × *g* for 5 min.
131. Firmly flip the plate to remove the supernatant.
132. Resuspend the cells in 100 μL Flow Buffer.
133. Transfer 50 μL of the dead cell control to a 1.5 mL reaction tube.
134. Incubate the dead cell control cells in the heat block at 67°C for 10 min to obtain a dead cell population.
135. Put the dead cell population back to the dead cell control well.
136. Add 2 μL of 7-AAD to the following samples and gently mix by pipetting up and down.
  a. Sample with all five antibodies.
  b. All FMO controls, except for the 7-AAD FMO control.
  c. Dead cell control.
137. Incubate the cells for 7 min at 20°C in the dark.
138. Resuspend cells in 150 μL Flow Buffer.
139. Analyze the cells with a flow cytometer.

**CRITICAL:** During the washing steps, the cell pellets are not always visible. Make sure quickly and firmly flip the plate to remove supernatant without losing the cell pellets.

**Table 2:** Antibody panel for characterizing cell population in ovarian tumor and ascites samples

Antibody target	Conjugate
CD326 (Epcam)	APC
CD45	PE-Cy7
CD90	PE
PDPN	BV605
CD31	BV421

### Cell death assay after drug treatment via flow cytometry

**Timing: 2 h**

This section describes the analysis of cell death after drug treatment of microspheroids using flow cytometry. The protocol allows to differentiate between epithelial cells (CD326+/CD45-), stromal cells (CD326-/CD45-), hematopoietic cells (CD326-/CD45+) and double positive cells (CD326+/CD45+), and determine the ratio of apoptotic cells within each cell population using 7-AAD and a marker for activated caspase-3/7. We use the Attune NxT Flow Cytometer for data acquisition. Adjust the protocol accordingly for other flow cytometer instruments.

**CRITICAL:** Keep the number of washing steps at minimum to avoid washing away dead cells.

140. Start after step 94.
141. Resuspend cells in Flow Buffer:
  a. Negative control (or untreated control) sample: 400 μL Flow Buffer.
  b. Drug treated sample: 100 μL Flow Buffer.
142. If required, count cells and check for cell clumps.
143. Add TruStain FcX to the cells (1:100) and gently mix by pipetting:
  a. Negative control sample: 4 μL TruStain FcX.
  b. Drug treated sample: 1 μL TruStain FcX.
144. Incubate for 10 min at 20°C.
145. Transfer the drug-treated sample to one well of a 96-well V-shaped microplate.
146. Divide the negative control (or untreated control) sample into seven 50 μL aliquots and transfer each aliquot into one well of a 96-well V-shaped microplate. For the dye panel described in this protocol, prepare the seven following flow sample wells:
  a. One sample with all markers: CD326 antibody, CD45 antibody, 7-AAD and Caspase-3/7 marker (7-AAD and Caspase-3/7 marker are added later).
  b. Four samples for each FMO control: one control for each of the two antibodies, one control for 7-AAD and one control for Caspase-3/7 marker.
  c. One sample for dead cell control.
  d. One sample for unstained control.
147. Prepare the 1:50 antibody dilutions with Flow Buffer according to Table 4. Prepare one dilution of #1 (all markers) for each of the treatment conditions: Negative control, drug treated sample.

**Table 4:** Antibody dilutions for flow cytometry

	Flow sample	Volume per antibody	Volume flow buffer	Total volume
#1	All markers with two antibodies: CD326, CD45	1.2 μL	57.6 μL	60 μL
#2	One FMO control for CD326 with CD45 antibody	1.2 μL	58.8 μL	60 μL
#3	One FMO control for CD45 with CD326 antibody	1.2 μL	58.8 μL	60 μL
#4	One FMO control for 7-AAD with two antibodies	1.2 μL	57.6 μL	60 μL
#5	One FMO control for Caspase-3/7 marker with two antibodies	1.2 μL	57.6 μL	60 μL
148. Centrifuge the plate at 500 × *g* for 5 min.
149. Firmly flip the plate to remove the supernatant.
150. Add 50 μL of each antibody dilution to the cells and add Flow Buffer to the dead cell control and to the unstained control.
151. Gently mix by pipetting up and down.
152. Incubate for 20 min at 4°C in the dark.
153. Add 100 μL Flow Buffer to the cells.
154. Centrifuge the plate at 500 × *g* for 5 min.
155. Firmly flip the plate to remove the supernatant.
156. Resuspend the cells in 100 μL Flow Buffer.
157. Transfer 50 μL of the dead cell control to a 1.5 mL reaction tube.
158. Incubate the dead cell control cells in the heat block at 67°C for 10 min to obtain a dead cell population.
159. Put the dead cell population back to the dead cell control well.
160. Add 0.1 μL of the CellEvent Caspase-3/7 green detection reagent to the following samples and gently mix by pipetting up and down:
  a. Sample with all markers (#1).
  b. All FMO controls (#2, #3 and #4), except for the Caspase-3/7 FMO control.
161. Incubate the cells for 45 min at 20°C in the dark before continuing with step 164.
162. Add 2 μL of 7-AAD to the following samples and gently mix by pipetting up and down:
  a. Sample with all markers (#1).
  b. All FMO controls (#2, #3 and #5), except for the 7-AAD FMO control.
  c. Dead cell control.
163. Incubate the cells for 7 min at 20°C in the dark.
164. Resuspend cells in 150 μL Flow Buffer.
165. Analyze the cells with a flow cytometer.

## Expected outcomes

In this protocol we describe the generation, culture, treatment and dissociation of patient-derived ovarian tumor microspheroids in an agarose-based multi-well system. In addition, we describe the analysis of the cellular composition and cell death for different cell types present in these microspheroids after cisplatin treatment via flow cytometry. The simultaneous culture of hundreds to thousands of microspheroids enables the recovery of single cells in high quantities and of high viability, essential for downstream single-cell analyses such as flow cytometry.

### Microspheroid morphology

**Figure 4:**
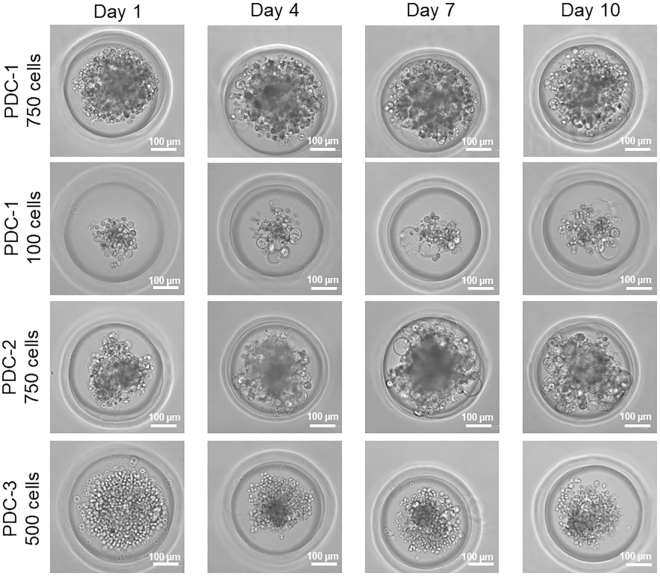
Live-cell images of microspheroids Exemplary brightfield images of patient-derived spheroids on day 1, 4, 7 and 10 after cell seeding. Spheroids are from 3 different patients and with different initial seeding densities. Scale bar = 100 μm.

The morphology of patient-derived microspheroids largely depends on the tissue of origin and the cell types that are included in the sample. It may also be modified by culture conditions, such as initial seeding density and culture medium composition. For ovarian cancer microspheroids, the morphology strongly varies between patient samples and depends on initial seeding density (Figure 4). In general, microspheroids can show a range of structural phenotypes, including cohesive spheroids with dense cores (Figure 4, first and third row) and less compact structures characterized by individual “satellite” spheroids or cells that are detached from the main spheroid (Figure 4, second row). Microspheroids may also display irregular borders or form a more spherical, well-defined shape. A mixture of these phenotypes is sometimes observed within the same sample. For instance, PDC-3 forms microspheroids with a compact core surrounded by individual “satellite” cells (Figure 4, fourth row). Additionally, some microspheroids develop cystic structures that appear as hollow, bubble-like features in brightfield images (Figure 4 first, second and third row).

### Viability of single cells

**Figure 5:**
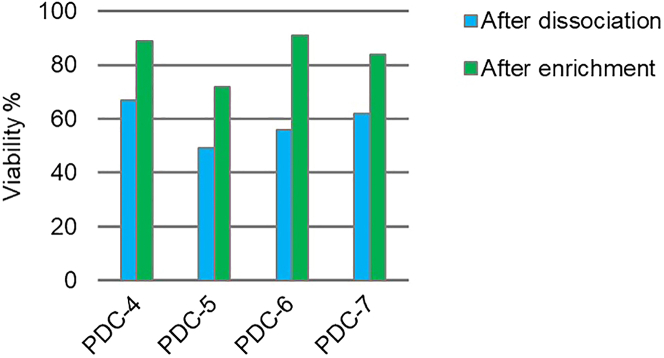
Single-cell viability of 4 different patient samples after spheroid dissociation and after enrichment for viable cells

The viability of dissociated single cells can vary between patient samples. The variability depends on different factors, including the tissue of origin, the quality of the sample used to generate the microspheroids, and the composition of cell types within the sample. For ovarian cancer microspheroids, we observed single cell viabilities ranging from around 50 % to up to 90 %. To increase viability of the single-cell suspension, we enriched for viable cell using the Dead Cell Removal Kit from Miltenyi (Figure 5).

### Cellular composition

**Figure 6:**
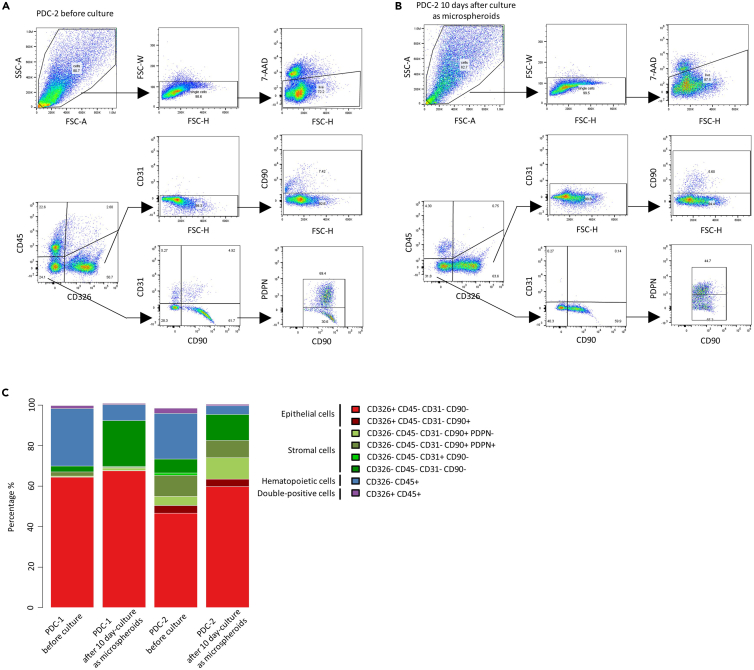
Single-cell composition of patient samples before and after culture as microspheroids for 10 days (A) and (B) Exemplary gating strategy of an ovarian cancer patient sample before and after microspheroid culture, including a doublet discrimination step and a live cell selection step. Arrows indicate the order of gating. (C) Cellular composition of two ovarian cancer patient samples before and after microspheroid culture.

Cells isolated from patient-derived ovarian tumor tissue or ascites fluid samples consist of a heterogenous mixture of different cell types, predominantly cancer cells, stromal cells (primarily fibroblasts) and hematopoietic cells.^15^^,^^16^ Our microspheroid models largely preserve the cellular diversity over time (Figures 6A and 6B). However, the microspheroid culture conditions presented in this protocol enrich for cancer and stromal cells, while the proportion of hematopoietic cells decreases over a culture period of ten days (Figure 6C). We further demonstrate that a rare CD90+ cancer cell subtype detected in one of the presented samples before culture remains present after 10 days in culture as microspheroids.

### Drug response

**Figure 7:**
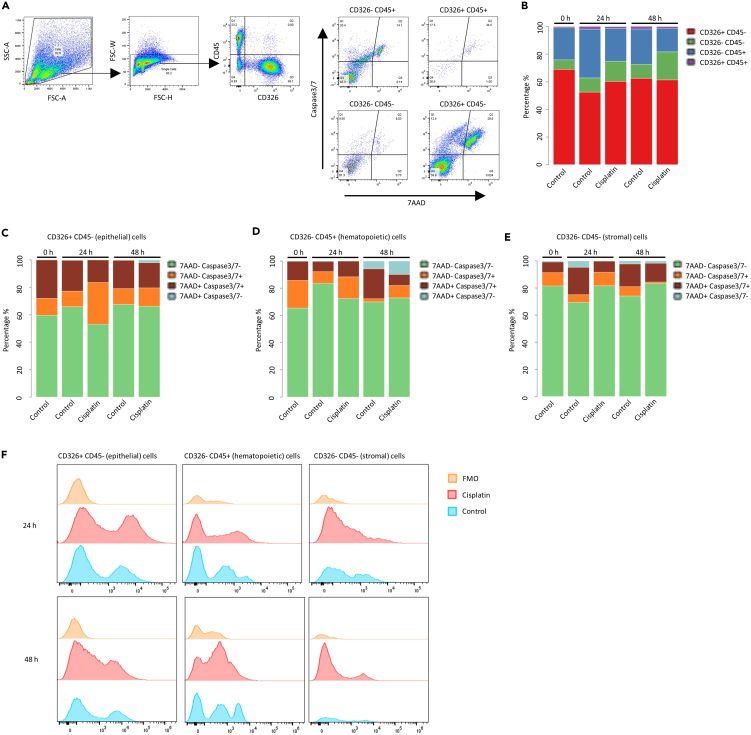
Single-cell drug response analysis via flow cytometry (A) Exemplary gating strategy of an ovarian cancer patient sample before cisplatin treatment, including a doublet discrimination step. Arrows indicate the order of gating. (B) Cellular composition before, 24 h after and 48 h after cisplatin treatment. (C) Cell death analysis of CD326+/CD45- (epithelial) cells. (D) Cell death analysis of CD326-/CD45+ (hematopoietic) cells. (E) Cell death analysis of CD326-/CD45- (stromal) cells. (F) Caspase-3/7 signal comparison for each of the cell populations.

Patient-derived ovarian tumor microspheroids show distinct cellular responses to cisplatin treatment as assessed via flow cytometry (Figure 7A). Within 48 h, microspheroids demonstrate a dynamic shift in cellular composition, characterized by an overall increase in CD326- CD45+ (hematopoietic) cells and CD326- CD45- (stromal) cells, and a corresponding decrease in CD326+ CD45- (epithelial) cells (Figure 7B, control). Following cisplatin treatment, each cell population displays a distinct response profile (Figure 7B): The proportion of hematopoietic cells decreases, stromal cells increase, and epithelial cells show a transient increase at 24 h with no marked difference at 48 h.

We further analyzed response to cisplatin for each cell population by measuring cell death with 7AAD and activated caspase-3/7, a marker for apoptosis. Epithelial and hematopoietic cells responded with a marked reduction in 7AAD- Caspase-3/7- (viable) cells and a corresponding increase in 7AAD- Caspase-3/7+ cells, indicating early apoptosis, 24 h post-treatment (Figures 7C and 7D). At 48 h, for both cell types the proportion of 7AAD+ Caspase-3/7- cells increased, indicating an increase in dead cells with no caspase-3/7 activity. In contrast, stromal cells responded to an increase in viable cells 24 h and 48 h post-treatment, likely reflecting relative enrichment due to loss of other cell populations (Figure 7E).

Caspase-3/7 signal intensity further confirms expected apoptotic kinetics after cisplatin treatment. Epithelial and hematopoietic cells demonstrate an increase in signal intensity at 24 h followed by a decline at 48 h (Figure 7F). In contrast, stromal cells did not show any change in caspase-3/7 signal intensity.

## Limitations

This protocol presents a scalable, cost- and time-efficient workflow for the simultaneous culture of hundreds of patient-derived tumor microspheroids, including the recovery and gentle dissociation into single cells for downstream molecular analysis, such as flow cytometry. While the protocol has been established with patient-derived ovarian tumor cells, it is important to note that the culture conditions, seeding densities, and dissociation methods might need optimization for other tumor entities or other cell types. Specifically, the Culture Medium has been optimized for ovarian cancer cell spheroids and might not be suitable for rare cell types present in ovarian tumors, such as immune cells. We recommend performing pilot experiments to test and optimize these aspects of the protocol for the specific cells of interest.

Another limitation is that not all cell types spontaneously form coherent spheroids and may instead remain as loose cell aggregates. Spheroid formation can be influenced by seeding density, with higher cell numbers promoting cell-cell interactions and the formation of coherent, dense structures. Medium composition and incubation duration can also affect spheroid formation. Optimizing these parameters may be necessary for cell types that do not readily form spheroids.

Finally, during the pipetting and transfer steps involved in recovering spheroids and single cells, there may be a loss of dead cells which could affect the results of drug response experiments. To mitigate this, we advise users to keep the number of washing steps to a minimum and to include sufficient technical replicates and appropriate controls to ensure reliable results.

## Troubleshooting

### Problem 1

Agarose multi-well dishes break during preparation (related to preparation of multi-well dishes).

### Potential solution

• Do not use agarose with ultra-low gelling temperature.
• Let the agarose-filled molds cool down at 20°C for at least 20 min, before releasing the multi-well dishes.
• Some practice might be necessary to prepare and release the agarose multi-well dishes. Before starting the experiment, prepare some dishes for practicing.

### Problem 2

Spheroids are too small or too big (related to seeding tumor cells to generate microspheroids).

### Potential solution

The size of the spheroids depends on the duration of culture, the culture conditions and can highly vary between samples. It is important to keep these factors in mind when deciding on the initial seeding density (Table 1). We recommend testing at least two different cell numbers before continuing the experiment. Note that the well diameter of the 16 × 16 array (12-256 cast mold) agarose dishes is smaller than the well diameter of a regular 384-well plate.

### Problem 3

Cells attach to the 12-well culture plate during culturing (related to seeding tumor cells to generate microspheroids and recovery and dissociation of microspheroids).

### Potential solution

It is possible that after seeding some cells diffuse out of the cell seeding chamber and attach to the culture plate bottom. This doesn’t affect the experiment if the cells stay attached during the recovery steps of the protocol. In case cells easily detach from the plate bottom, e.g., due to lose adherence to the plastic or due to treatment causing detachment, the agarose multi-well dish should be transferred to a new 12-well culture plate before inverting the dish and releasing the spheroids.

### Problem 4

Cells do not form compact spheroids, but remain as loose aggregates that easily dissociate into single cells during recovery (related to seeding tumor cells to generate microspheroids and recovery and dissociation of microspheroids).

### Potential solution

Spheroid formation can strongly vary between patient samples and cell types. Some samples may form only loose cell aggregates rather than cohesive, compact spheroids. In certain cases, spheroid formation can be enhanced by optimizing the culture conditions, such as increasing the initial seeding density, extending incubation time or adjusting the culture medium composition to support cell-cell interactions.

### Problem 5

Spheroids are very compact and do not dissociate into single cells within 30 min (related to recovery and dissociation of microspheroids).

### Potential solution

Spheroid formation can strongly vary between patient samples and cell types. Some samples form tight aggregates that are difficult to dissociate into single cells. We do not recommend a longer dissociation time than 30 min, unless it has been shown to not affect cell integrity. Try another dissociation enzyme, such as Accutase, Accumax, papain or collagenase, instead. Make sure the dissociation process does not affect the viability of cells or the expression of surface proteins of interest.

### Problem 6

The proportion of viable cells is low after dissociation (related to recovery and dissociation of microspheroids).

### Potential solution

If downstream molecular analyses require a high proportion of viable cells (e.g., single-cell RNA sequencing) the cell suspension can be enriched for viable cells using the Dead Cell Removal Kit as described in the section enrichment for viable cells (optional). To increase the efficiency of the enrichment, the procedure can be repeated over a second column. If microbead-based enrichment is still not sufficient, viable cells can be sorted using fluorescence-activated cell sorting and a viability marker.

## Resource availability

### Lead contact

Requests for further information and resources should be directed to and will be fulfilled by the lead contact, Brinton Seashore-Ludlow (brinton.seashore-ludlow@ki.se).

### Technical contact

Questions about the technical specifics of performing the protocol should be directed to the technical contact, Mariam Haffa (mariam.haffa@ki.se).

### Materials availability

This study did not generate new reagents.

### Data and code availability

This study did not generate or analyze new datasets or codes.

## Acknowledgments

This work was supported by the RED Equipment grant award from 10.13039/501100009252SciLifeLab Campus Solna 2022, the 10.13039/501100004359Swedish Research Council (2021-03420), 10.13039/501100007232Radiumhemmets forskningsfonder 231362, and by the German Cancer Aid (Deutsche Krebshilfe, Mildred-Scheel-Post doktoranden programm). The graphical abstract was created using Biorender.com.

## Author contributions

M.H., K.J., and B.S.-L. developed the protocol. M.H., K.J., G.G., and R.H.-G.Ö. designed and performed the experiments. M.H. and K.J. analyzed the data. E.W., J.F., and U.J. provided patient samples. M.H. and K.J. wrote the manuscript. B.S.-L., O.-P.K., and M.H. acquired the funding. All authors read and approved the manuscript.

## Declaration of interests

O.-P.K. is a board member and co-founder of Sartar and an advisor to the Knut and Alice Wallenberg Foundation, Novo Nordisk Foundation, and Sitra.

## Declaration of generative AI and AI-assisted technologies in the writing process

During the preparation of this work, the authors used ChatGPT (GPT-5.1 OpenAI) in order to improve readability of the text. After using this tool, the authors reviewed and edited the content as needed and take full responsibility for the content of the published article.
